# JUUL the heartbreaker: Twitter analysis of cardiovascular health perceptions of vaping

**DOI:** 10.18332/tid/130961

**Published:** 2021-01-08

**Authors:** Traci Hong, Jiaxi Wu, Derry Wijaya, Ziming Xuan, Jessica L. Fetterman

**Affiliations:** 1 College of Communication, Boston University, Boston, United States; 2 Department of Computer Science, Boston University, Boston, United States; 3 School of Public Health, Boston University, Boston, United States; 4 Evans Department of Medicine, Boston University School of Medicine, Boston, United States; 5 Whitaker Cardiovascular Institute, Boston University, Boston, United States

**Keywords:** electronic cigarettes, e-cigarettes, JUUL, health perceptions, Twitter

## Abstract

**INTRODUCTION:**

The public most frequently associates tobacco use solely with pulmonary health risks, despite heart disease being the leading cause of death in smokers. The health perceptions of e-cigarettes, especially cardiovascular health, have not been well studied. We aimed to evaluate the prevalence and health perceptions of tweets related to cardiovascular, pulmonary, and brain health – three organ systems for which tobacco use is a major disease risk factor.

**METHODS:**

We examined the cardiovascular, pulmonary, and brain health perceptions of vaping and JUUL on Twitter, followed by a content analysis of tweets pertaining to the cardiovascular risks. A Twitter firehose API scraped about 6.2 million publicly available tweets from 2015–2019 that contained vaping-related terms, and a separate dataset of about 1.9 million tweets that contained the term JUUL. A quantitative content analysis (n=2145) of tweets was subsequently conducted to assess the health perceptions of vaping and JUUL. Two trained coders independently assessed the posts and Twitter profiles to determine age (<18 or ≥18 years), sex, race, sentiment towards JUUL, and vaping-related topics.

**RESULTS:**

The majority of tweets containing vaping or JUUL-related terms did not also contain cardiovascular, pulmonary, or brain health terms (97.99% and 96.67%, respectively). Multiple linear regression analysis showed that youth (<18 years), females, non-White individuals, mention of a flavor, and mention of cardiovascular health harm words were associated with more positive sentiments towards JUUL. Pearson’s chi-squared analyses indicated that youth were more likely to mention a JUUL flavor. Females and youth were more likely to reference cardiovascular terms with humor.

**CONCLUSIONS:**

The cardiovascular health risks of vaping are not fully recognized by the public. Vulnerable populations such as youth and females reference JUUL with cardiovascular-related words that downplay the severity of tobacco as a major risk factor for cardiovascular disease.

## INTRODUCTION

JUUL entered the US market in 2015 and quickly came to dominate e-cigarette market sales. From 2015 to 2019, e-cigarette use steadily increased among youth and young adults, and e-cigarette use among high school students increased by 135% from 2017 to 2019, which has largely been attributed to the introduction of JUUL^1,2^. JUUL is the most popular e-cigarette among youth with about 59% of high school e-cigarette users reporting JUUL use^1^. Because of its small size and nearly absent aerosol cloud, JUUL use is discreet allowing youth to sneak a ‘hit’ in the middle of classes, when they would otherwise be easily caught. Unlike the earlier generations of e-cigarettes, JUUL utilizes a nicotine salt that increases nicotine absorption in the lung, allowing circulating nicotine levels to reach similar levels achieved using combustible cigarettes^3,4^.

E-cigarettes are often marketed as reduced-risk tobacco products^5^; although emerging evidence suggests that e-cigarette use is associated with alterations in cardiovascular function and pulmonary symptoms^6,7^. Products that are marketed as reduced-risk can have a direct impact on consumer perceptions and behaviors, which may lead to use among vulnerable populations. The risk of experimentation and initiation of tobacco use is associated with decreased perception of harm, particularly among youth^8-10^. Currently, the disease-specific perceptions associated with vaping and newer products like JUUL, particularly among youth, are poorly understood.

Social media are platforms for public communication that tobacco researchers can use to gauge health perceptions and behaviors. About 45% of youth report being constantly online^11^. Twitter is one of the most popular platforms with 186 million active daily users^12^. We sought to evaluate the prevalence and health perceptions of vaping-containing tweets related to cardiovascular, pulmonary, and brain health. We then subsequently conducted a quantitative content analysis of tweets that pertained to cardiovascular health and JUUL, and evaluated the demographics of the tweet poster.

## METHODS

### Sample

We collected all publicly available Twitter posts (about 1.9 million) that contained the term ‘JUUL’ from 1 January 2015 to 29 November 2019, using Crimson Hexagon, which utilizes the Twitter firehose API. The time period encompasses the introduction of JUUL to the US market (2015) and the 2019 E-cigarette or Vaping Product Use-Associated Lung Injury (EVALI) cases^7^. As a point of comparison, we also collected publicly available Twitter posts (about 6.2 million) that contained at least one vaping term (vaping, vape, vaper, vapers, vapin, vaped evape, vaporing, e-cig, ecig, e-pen, epen, e-juice, ejuice, e-liquid, eliquid, cloud chasing, cloudchasing, vapepen, vape pen). The datasets are not mutually exclusive with a 3.9% overlap in tweets. All tweets are written in English and originated from the US.

**Figure 1:**
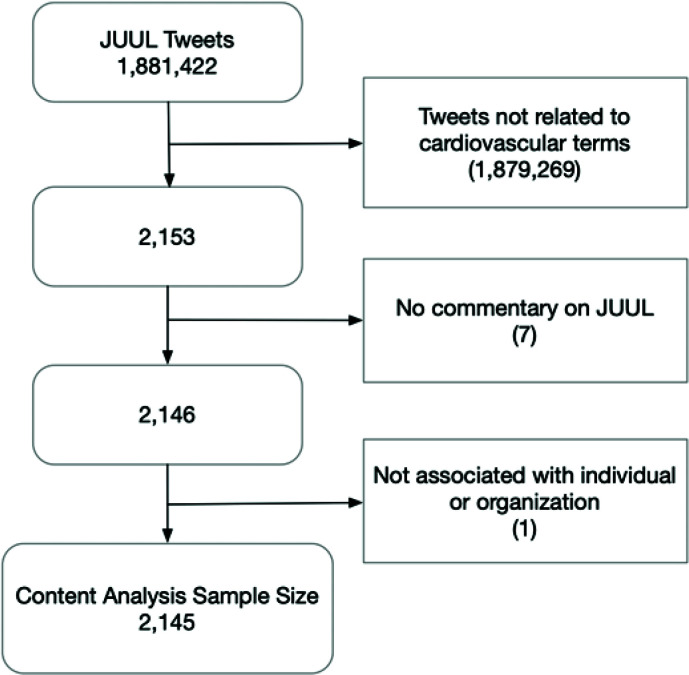
Flow chart of the study sample for content analysis

For both the vaping and JUUL datasets, we used string matching to determine the number of tweets containing keywords associated with the three major organ systems impacted by tobacco: 1) pulmonary (lungs, lung, breath, breathe, breathing, pulmonary, asthma, COPD, chest); 2) brain (brain); and 3) cardiovascular (cardiac, cardiovascular, heart disease, heart health, heart problem, hypertension, high blood pressure, diabetes, heart attack, myocardial infarction, heart failure, cardiac arrest, chest pain, blood clot, cholesterol, blood sugar, aneurysm, stroke). To conduct an in-depth analysis of how cardiovascular health is discussed with regard to JUUL, we conducted a quantitative content analysis of the 2153 tweets that contained at least one of the pre-identified cardiovascular terms. We discarded posts that contained no commentary on JUUL (n=7), and posts that were not associated with an individual or an organization (n=1), resulting in a final sample of 2145 posts. The tweet was the unit of analysis. Figure 1 shows the flow chart of the study sample for the content analysis.

### Coding procedure and inter-coder reliability

Two trained graduate student coders independently examined the tweets to determine the sentiment towards JUUL (negative, neutral, positive). Tweet type (e.g. news, advertisement or commentary) and an indication of a flavor (mint/menthol, tobacco, fruit, other) were coded. In the statistical analyses, flavor was coded as a dichotomous variable (presence/absence). We coded the presence/absence of the following two content areas: humor and harm. An example of humor is ‘when my Juul stops working or I lose it I have a heart attack’. Harm pertains to tweets linking vaping with deleterious cardiovascular health effects. An example of harm is: ‘went through a whole box of juul pods last night. I need a stop before I have a stroke’.

The coders examined the profile pictures, Twitter handles, posted images/videos, and the biographical descriptions to determine demographic data including sex (male, female), race (White, non-White, unknown), and age (<18 or ≥18 years), and if the tweet was associated with an actual human being (individual account vs organization). Age was coded ≥18 years in any instances where there was ambiguity. Organization accounts were not coded for age, sex, or race. Frequencies of variables in the content analysis are shown in the Supplementary file, Table S1.

To assess inter-coder reliability (Cohen’s Kappa)^13^, 201 (about 10%) posts were randomly selected and both coders, blind to the study purpose, independently coded the selected posts. The inter-coder reliabilities for the 9 variables in our study were: sex (0.99), race (0.80), age (0.72), individual/organization account (1.0), tweet type (0.72), humor (0.88), sentiment (0.73), flavor (1.0), and harm (0.97). Kappa values of 0.61–0.80 are considered substantial, and values ≥0.81 are considered almost perfect agreement^14^. Upon determination that data coding was reliable, the trained coders then independently coded the remaining posts. The study was approved by Boston University Institutional Review Board.

### Statistical analysis

Multiple linear regression was used for predicting sentiment toward JUUL from sex, race, age, flavor, and harm. Pearson’s chi-squared test and calculated odds ratios were used to examine the association between demographics (sex, race, and age) with flavor, use of cardiovascular terms in humor, and harm.

## RESULTS

**Figure 2:**
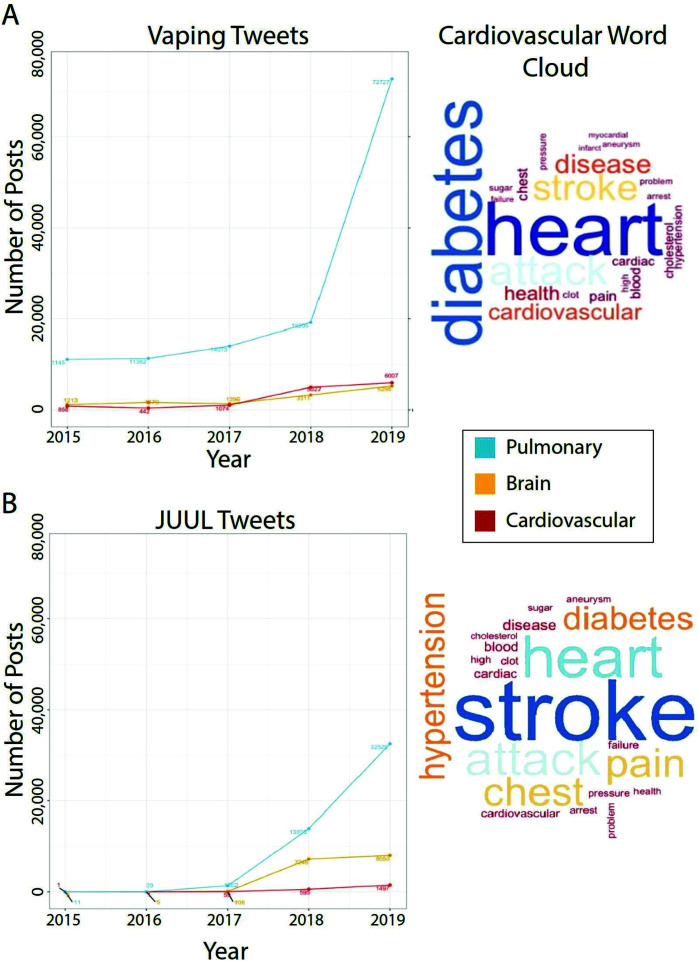
Prevalence of tweets on vaping (A) and JUUL (B) containing keywords associated with pulmonary, brain, and cardiovascular health from 2015–2019 (Left). Corresponding word clouds illustrate the frequency of each cardiovascular term (larger size indicates greater frequency, Right).

Figure 2 gives the prevalence of tweets-containing terms related to cardiovascular, pulmonary, and brain systems from 2015 to 31 November 2020 for tweets that contained terms related to ‘vaping’ (Figure 2A) and ‘JUUL’ (Figure 2B). The majority of tweets containing vaping or JUUL-related terms did not also contain cardiovascular, pulmonary, or brain health terms (97.99% and 96.67%, respectively). The frequency of cardiovascular terms with ‘vaping’ or ‘JUUL’ are provided in the Supplementary file, Table S2. For vaping, tweets related to pulmonary health (n=128533; 2.1%) were the most prevalent, followed by cardiovascular (n=13408; 0.2%) and brain (n=12894; 0.2%). For JUUL, tweets related to pulmonary health (n=47876; 2.5%) were the most prevalent, followed by brain (n=15415; 0.8%) and cardiovascular health (n=2153; 0.1%). The steep increase in tweets containing pulmonary health words from 2018 to 2019 likely reflects the emergence of EVALI. The most frequent cardiovascular terms were stroke (n=771), heart attack (n=399), chest pain (n=319), and hypertension (n=247).

We evaluated the predictors of positive sentiment towards JUUL. Multiple regression analysis indicates that sex, age, race, harm, and flavor predict positive sentiment towards JUUL [F(5,1751)=283.22; p<0.001]. Females (β=0.267, p<0.001), age <18 years (β=0.127, p=0.049), non-White (β=0.171, p<0.001), and mention of flavor (β=0.186, p=0.018) were associated with a more positive sentiment towards JUUL. Recognition of cardiovascular health harm of JUUL inversely predicted positive sentiment, (β=-1.107, p<0.001), with an overall model fit of R^2^=0.45.

Using Pearson’s chi-squared test, we evaluated the association between the demographics of the tweet author with the JUUL-related tweet content. The age of the author was associated with mention of a flavor in the tweet [χ^2^(1)=5.113, p=0.024]. Youth were 2.27 times more likely than adults to tweet about a JUUL flavor. Both sex [χ^2^(1)=76.274, p≤0.001] and age [χ^2^(1)=6.914, p=0.009] were associated with the use of humor in reference to a cardiovascular term. Specifically, females were 2.58 times more likely than males to use humor in reference to a cardiovascular term and youth were 1.7 times more likely than an adult to use humor in reference to a cardiovascular term. Sex was associated with the perception of harmful cardiovascular health effects of vaping [χ^2^(1)=17.271, p<0.001]. Females were 33.6% less likely than males to associate vaping with harmful cardiovascular health effects.

## DISCUSSION

Twitter discussions of the cardiovascular health effects of JUUL trail pulmonary and brain discussions, which is consistent with the public perception that primarily attributes tobacco-related health effects to the lungs. Vulnerable populations such as non-Whites, youth, and females expressed more positive sentiments about JUUL. Tweets by youth were more likely to reference flavors, consistent with previous studies showing that flavored tobacco use is higher among youth than adults^15^. Our study reinforces previous findings that women are less aware of the adverse effects of tobacco use on cardiovascular health^16^. Strikingly, we found that females and youth are more likely to use vaping-related cardiovascular terms with humor, which downplays the harmful effects of vaping on cardiovascular health. In addition, the tweets from organizations represented only 7% of the body of tweets on JUUL and cardiovascular health, indicating that organizations need to play a greater role in disseminating evidence-based messages that link vaping with cardiovascular health effects.

Current studies evaluating the health perceptions of vaping and JUUL have focused on tobacco product users or adults^17,18^, and perceptions of overall health risks^18,19^, rather than diseases impacting specific organ systems. Ever JUUL users also report the perception that JUUL use is less harmful than use of all other tobacco products^18^. Similarly, an online survey found that 39% of youth perceived JUUL as less harmful than combustible cigarettes^19^. In a national survey, health perceptions of e-cigarettes differed by flavor category with high school students perceiving fruit flavors to be less likely to lead to lung cancer^20^. Our study shows that the discussion of the health effects of vaping and JUUL use are limited on a popular social media platform and that vulnerable populations use cardiovascular health terms primarily in the context of humor. The positive sentiment and absence of discussion on the negative health effects of vaping may contribute to increased experimentation and use among youth, non-Whites, and women.

### Limitations

Our study has a number of limitations. Twitter users may not be representative of the general population with young adults being over-represented compared to adults. Tweets are by design parsimonious in words and thus may not capture the full sentiment regarding JUUL and vaping with cardiovascular health. Although the inter-coder reliability was high, we are limited to the images and content of the Twitter profiles in evaluating the demographic characteristics of tweet authors. Due to the cross-sectional study design and lack of information regarding the tobacco use by the tweet authors, we are unable to evaluate whether the health perceptions impact future product use. The proportion of the tweets related to vaping or JUUL that contained pulmonary, cardiovascular, or brain health terms was small and may limit the conclusions that can be drawn.

## CONCLUSIONS

The small proportion of tweets related to the health risks of vaping and JUUL use raises concerns regarding whether the public recognizes the potential health risks associated with vaping. Among the tweets pertaining to health perceptions, pulmonary risks are most frequently referenced. Vulnerable populations reference JUUL with cardiovascular-related words in a context that downplays the severity of tobacco as a major risk factor for cardiovascular health. Our study highlights the need for public education to increase the awareness of the health harms, particularly cardiovascular effects, associated with tobacco use.
