# Supplementary material for: JUUL the heartbreaker: Twitter analysis of cardiovascular health perceptions of vaping
Source: Tob Induc Dis. 2021 Jan 8;19:01. doi: 10.18332/tid/130961 (PMC7791365; doi:10.18332/tid/130961)
Supplement: Supplementary file 1 [file TID-19-01-s1.pdf]

Table S1. Frequencies of Variables in Content Analysis

	Frequency (%)
**Content Analysis Coding Variable**	
**Account Type**	
Individual	1989 (93)
Organization	156 (7)
**Tweet Type**	
News	346 (16)
Advertisement	1 (0.04)
Commentary	1784 (84)
**Humor**	
No	1676 (79)
Yes	455 (21)
**Harm**	
No	772 (36)
Yes	1359 (64)
**Flavor**	
Mint/Menthol	71 (3)
Tobacco	1 (0.05)
Fruit	2 (0.09)
Other	5 (0.2)
None	2052 (96)
**Sentiment**	
Negative	1,316 (62)
Neutral	301 (14)
Positive	514 (24)
**Sex**	
Male	1012 (54)
Female	872 (46)
**Race**	
White	1332 (74)
Non-white	458 (25)
**Age**	
< 18	108 (6)
≥ 18	1686 (94)

Table S2. Frequency of Cardiovascular Words Appearing in Tweets with Vaping-Related Terms and JUUL

	Vaping Frequency (%)	JUUL Frequency (%)
**Cardiovascular Term**		
Stroke	2426 (18)	771 (36)
Heart Attack	2434 (18)	399 (18)
Chest Pain	383 (3)	319 (15)
Hypertension	73 (0.5)	247 (11)
Diabetes	4798 (36)	242 (11)
Heart Disease	1373 (10)	62 (3)
Cardiovascular	1109 (8)	30 (1)
Blood Clot	118 (0.9)	26 (1)
Cardiac	298 (2)	24 (1)
Heart Failure	40 (0.3)	22 (1)
Cardiac Arrest	106 (0.8)	17 (0.8)
High Blood Pressure	96 (0.7)	17 (0.8)
Heart Problem	83 (0.6)	14 (0.6)
Aneurysm	51 (0.4)	11 (0.5)
Heart Health	1079 (8)	10 (0.4)
Blood Sugar	55 (0.4)	8 (0.4)
Cholesterol	130 (1)	7 (0.3)
Myocardial Infarction	18 (0.1)	1 (0.05)

© 2021 Hong T. et al.
